# Efficacy of acupuncture for the treatment of Parkinson’s disease-related constipation (PDC): A randomized controlled trial

**DOI:** 10.3389/fnins.2023.1126080

**Published:** 2023-02-13

**Authors:** Ying-Jia Li, Ian-I Leong, Jing-Qi Fan, Ming-Yue Yan, Xin Liu, Wei-Jing Lu, Yuan-Yuan Chen, Wei-Qiang Tan, Yu-Ting Wang, Li-Xing Zhuang

**Affiliations:** ^1^Clinical Medical College of Acupuncture-Moxibustion and Rehabilitation, Guangzhou University of Chinese Medicine, Guangzhou, China; ^2^The First Clinical College, Guangzhou University of Chinese Medicine, Guangzhou, China; ^3^Department of Acupuncture and Moxibustion, The First Affiliated Hospital of Guangzhou University of Chinese Medicine, Guangzhou, China

**Keywords:** Parkinson’s disease, constipation, acupuncture, sham acupuncture, non-motor symptom, randomized controlled trial

## Abstract

**Objective:**

To evaluate the efficacy of acupuncture in treating Parkinson’s disease-related constipation (PDC).

**Materials and methods:**

This was a randomized, controlled trial in which patients, outcome assessors, and statisticians were all blinded. Seventy-eight eligible patients were randomly assigned to either the manual acupuncture (MA) or sham acupuncture (SA) groups and received 12 sessions of treatment over a 4-week period. Following treatment, patients were monitored until the eighth week. The primary outcome was the change in weekly complete spontaneous bowel movements (CSBMs) from baseline after treatment and follow-up. The Constipation Symptom and Efficacy Assessment Scale (CSEAS), the Patient-Assessment of Constipation Quality of Life questionnaire (PAC-QOL), and the Unified Parkinson’s Disease Rating Scale (UPDRS) were used as secondary outcomes.

**Results:**

In the intention-to-treat analysis, 78 patients with PDC were included, with 71 completing the 4-week intervention and 4-week follow-up. When compared to the SA group, weekly CSBMs were significantly increased after treatment with the MA group (*P* < 0.001). Weekly CSBMs in the MA group were 3.36 [standard deviation (SD) 1.44] at baseline and increased to 4.62 (SD, 1.84) after treatment (week 4). The SA group’s weekly CSBMs were 3.10 (SD, 1.45) at baseline and 3.03 (SD, 1.25) after treatment, with no significant change from baseline. The effect on weekly CSBMs improvement in the MA group lasted through the follow-up period (*P* < 0.001).

**Conclusion:**

Acupuncture was found to be effective and safe in treating PDC in this study, and the treatment effect lasted up to 4 weeks.

**Clinical trial registration:**

http://www.chictr.org.cn/index.aspx, identifier ChiCTR2200059979

## 1. Introduction

Parkinson’s disease (PD) is a progressive neurodegenerative disease with a multifaceted etiology (Bloem et al., 2021). In addition to classical motor features, PD is also characterized by extremely complex non-motor symptoms (NMS) (Armstrong and Okun, 2020). The pathophysiological process of PD includes α- synuclein aggregation in the nigra and striatum, neuroinflammation, and mitochondrial dysfunction. The complexity of these intertwined pathophysiological processes and the resulting heterogeneity of clinical symptoms will require a targeted method (Vijiaratnam et al., 2021). Constipation, an integral part of NMS, has received greater attention in the last 15 years (Mukherjee et al., 2016). Reduced defecation frequency, excretion of hard stools, prolonged defecation, and sensations of incomplete evacuation and bloating are the most common symptoms of Parkinson’s disease-related constipation (PDC) (Sung et al., 2014). Based on the Rome III criteria, the estimated prevalence of PDC ranges from 46.83 to 59.6% (Chen et al., 2015), with higher rates reported in Asia (Gan et al., 2018). Current evidence suggests that abnormally folded α-syn aggregates from Lewy bodies and neurites in the enteric nervous system play a role in the pathogenesis of PD (Cersosimo et al., 2013; Mukherjee et al., 2016). Some PD patients experience constipation years before motor symptoms appear (Dogra et al., 2022). With colon motility altered, constipation affects patients at any stage of PD and greatly reduces the efficacy of levodopa, making PD management difficult (Lesser, 2002; Cersosimo and Benarroch, 2008). As PD progresses, the increasing prevalence of constipation negatively impacts the quality of life of patients, who are more susceptible to sleep disturbances and depression (Yu et al., 2018). There is no doubt that constipation is developing into a significant problem in parallel with motor symptoms (Schapira et al., 2017).

Currently, increasing dietary fiber and fluid intake is commonly used to treat PDC (Stern and Davis, 2016). When lifestyle changes are unhelpful, probiotics and prebiotic fiber, macrogol, and lubiprostone may be used, though these have limited evidence for treating PDC (Rossi et al., 2015). Constipation in PD patients is recurrent and refractory, which requires continuous and long-term treatment (Barboza et al., 2015). Despite the fact that different prokinetics and laxatives are commonly used, they might not be the ideal choice for the long-term treatment of PDC because side effects, including cramping and bloating, could occur after continuous use (Mozaffari et al., 2020). In addition, as the condition develops, patients with PD must move to higher doses of anti-Parkinson’s drugs to relieve motor symptoms (Chou et al., 2018). With the sheer number of oral medications available, they are more likely to choose non-pharmacological treatments for constipation (Seppi et al., 2019).

According to the American Parkinson’s Association (APA), the management of Parkinson’s disease is a multi-disciplinary effort that involves not only pharmacological but also non-pharmacological interventions (Cutson et al., 1995; Goldman and Guerra, 2020). Acupuncture is a common non-pharmacological treatment for improving gastrointestinal and neurological symptoms (Kwon et al., 2021; Fan et al., 2022a; Yao et al., 2022). Studies have shown that acupuncture can improve gastrointestinal and neurological symptoms while also relieving emotional symptoms (Wang et al., 2022a; Ying-Jia Li et al., 2022). However, it has also been questioned if this is a result of the placebo effects of acupuncture. Therefore, appropriate blinded and high-quality clinical studies are required to determine whether acupuncture has specific effects in treating PDC. High-quality clinical evidence on the efficacy of acupuncture for PDC is lacking (Zeng and Zhao, 2016). Two previous meta-analyses have discussed the effect of acupuncture on PDC; however, the conclusions of the two studies were inconsistent, mainly due to the significant heterogeneity and small sample size of the existing studies (Cheng, 2017; Li et al., 2022). As a result, we created a randomized, single-blind clinical trial with sham acupuncture (SA) as a control to evaluate the efficacy of manual acupuncture (MA) for PDC.

## 2. Study design and materials and methods

### 2.1. Recruitment and ethical review

Between May 2022 and November 2022, the patients with PDC were recruited in the outpatient department of Parkinson’s disease at the First Affiliated Hospital of Guangzhou University of Chinese Medicine. All patients signed the informed consent form after being fully informed of the study’s purpose and process.

This clinical trial has been approved by the Ethics Committee of the First Affiliated Hospital of Guangzhou University of Chinese Medicine (Ethics number: K-2022-005) and has been registered in China Clinical Trial Center (ChiCTR2200059979).

### 2.2. Study design and patients

The total trial period of this randomized, single-blind clinical trial was 9 weeks, consisting of a baseline phase (−1 to 0 week), an intervention phase (1 to 4 weeks), and a follow-up phase (4 to 8 weeks). We used the Standards for Reporting Interventions in Clinical Trials of Acupuncture (STRICTA) and the Consolidated Standards of Reporting Trials (CONSORT) statements as study guidelines (MacPherson et al., 2010).

The enrolled patients met the following inclusion and exclusion criteria after being evaluated by an experienced physician. Inclusion criteria included a diagnosis of PD (according to the Movement Disorder Society’s revised clinical diagnostic criteria for Parkinson’s disease in 2015) (Postuma et al., 2015) and functional constipation (according to the Rome IV diagnostic criteria) (Palsson et al., 2016), age between 35 and 80 years, Hoehn-Yahr grade ≤ 3, no medications taken within 2 weeks that may affect gastrointestinal function (such as prucalopride and probiotics), no participation in other clinical trials within 1 month, voluntary engagement in this study, ability to sign the informed consent, and cooperation in the completion of the bowel diary and scale filling. Exclusion criteria included organic lesions of the digestive system (such as intestinal adhesions, obstructions, tumors, or malformations in the gastrointestinal tract), a history of abdominal or anorectal surgery that may affect intestinal transit, systemic diseases that may affect the dynamics of the digestive tract (such as diabetes and hyperthyroidism), serious life-threatening diseases (such as severe cardiovascular diseases and malignant tumors), skin lesions that were inappropriate for needling, the viscose allergy that prevented acupuncture device attachment, and pregnant or lactating women.

### 2.3. Randomization and blinding

We used simple randomization for grouping. An independent researcher generated the random number tables by SPSS 26.0 and assigned the patients to the MA and SA groups in a 1:1 ratio according to the random numbers. The uniformly shaped cards with treatment conditions were placed inside the opaque envelopes, which were opened before treatment.

Patients, outcome assessors, and statisticians were all blinded in this study. To prevent communication between two groups, the therapy sessions were scheduled at different times of the day. To assure blindness, patients were asked to wear an eye mask during the treatment. All physicians performed the treatments in accordance with standardized procedures to maintain consistency of study. As for unblinding, patients were informed of their grouping after treatment and received the type of acupuncture after a follow-up.

For control, we used a special acupuncture device that was designed by our team members (patent number ZL202121352221.7) (Wang et al., 2022b). As shown in Supplementary Figure 2 (Supplementary material 1), this acupuncture device consisted of a base and a cannula, classified into two types. In the MA group, the base had a longitudinal opening at the bottom, through which ordinary acupuncture needles could be pierced into the skin. In the SA group, the base had no opening at the bottom, so special flat-head needles could not pierce the skin. The special flat-head needle was designed to be shorter in length so that the exposed needle body length is the same in both groups, ensuring uniformity of appearance throughout operation. Besides, the cannula’s angle was made to be flexible to accommodate different needling locations.

### 2.4. Interventions

Four licensed physicians with more than 3 years of clinical experience performed the treatments. To maintain consistency of operation, all physicians received initial training on the treatment’s standardized procedures. Throughout the study, patients in both the MA and SA groups were required to maintain effective therapeutic doses of anti-Parkinson’s drugs at levels similar to their baseline. Patients with any dosage adjustment, whether an increase, decrease, or adjustment, were dropped. When patients did not have complete spontaneous bowel movements (CSBMs) for more than three consecutive days or experienced intolerable symptoms such as bloating due to constipation, they were allowed to use emergency medications (including lactulose and glycerin enemas) under medical supervision with detailed documentation. The first bowel movement after each use of emergency medication could not be recorded in the results.

#### 2.4.1. Acupuncture methods

All patients received 12 sessions of treatment, three times a week (Tuesday, Thursday, and Saturday), for a period of 4 weeks. Each session lasted 30 min. All acupoints were selected based on traditional Chinese medicine theory and previous articles on PD and constipation (Liu et al., 2016; Li et al., 2019; Nazarova et al., 2022). The acupoints used by the MA and SA groups included Sishenzhen (four acupoints, consisting of GV21, GV19, and next to GV20 1.5 cun bilateral), GV24 (Shenting), GV29 (Yintang), ST25 (Tianshu), CV4 (Guanyuan), and ST37 (Shangjuxu). All acupoints were taken bilaterally and positioned in accordance with the National Standard of the People’s Republic of China (GB/T12346-2006). The location, insertion depth, and direction of the acupoints used in this study were all listed in the Supplementary Table 3 in Supplementary material 1.

The patient was required to put on an eye mask while receiving treatment. The MA group’s patients used acupuncture devices with a longitudinal opening at the bottom. In the SA group, sham acupuncture devices without an aperture were employed. Physicians precisely located the acupoints and sterilized the skin around them. Then, in the MA group, physicians pierced disposable sterile acupuncture needles into the skin (0.30 mm × 25 mm, 0.30 mm × 40 mm, Suzhou Tianxie Medical Supplies Co., Ltd., Suzhou, China), and in the SA group, physicians used sterile flat-head acupuncture needles without piercing the skin (0.30 mm × 10 mm, Guangzhou Suixin Medical Supplies Co., Ltd., Guangzhou, China). In both groups, the needles were left in for 30 min before being taken out.

### 2.5. Outcomes

Age, sex, duration of disease, and equivalent daily dose of levodopa were assessed at baseline. From the beginning of the baseline phase to the end of the follow-up phase, patients were requested to complete an electronic bowel diary, which was supervised by an independent outcome assessor.

#### 2.5.1. Primary outcome

The primary outcome in this study was the number of weekly CSBMs. Weekly CSBMs were collected at baseline (week 0), post-treatment (week 4), and follow-up (week 8), and differences from baseline levels were compared at week 4 and week 8.

#### 2.5.2. Secondary outcomes

Secondary outcomes included the Constipation Symptom and Efficacy Assessment Scale (CSEAS) (including the six dimensions of difficulty, Bristol, time, incompleteness, frequency, and bloating), the Patient-Assessment of Constipation Quality of Life questionnaire (PAC-QOL), and the Unified Parkinson’s Disease Rating Scale (UPDRS). These were assessed at baseline, post-treatment, and follow-up.

#### 2.5.3. Safety assessments and evaluation of blinding

Acupuncture-related adverse effects were described in detail. When adverse effects occurred, based on the patient’s wishes, the physician assessed whether the patient was still appropriate for continued research involvement.

To assess the effect of blinding, at the end of the study, we asked patients to guess their assignment status and whether they believed the acupuncture needle pierced the skin.

### 2.6. Data monitoring and analysis

Based on the results of the pilot study we conducted, the change in weekly CSBMs from baseline was 0.964 [standard deviation (SD), 1.726] times in the MA group and −0.321 (SD, 1.310) times in the SA group. According to the calculation of PASS version 15.0, 31 patients would be needed in each group, with a two-sided significance level of 5% and a power of 90%. Given the estimated 20% loss-to-follow-up rate, we planned to enroll 78 patients in the study (39 patients in each group).

An independent statistician used SPSS 26.0 to conduct all analyses. We used the intention-to-treat (ITT) principle, regardless of whether the patients completed all assigned intervention sessions. Continuous variables were expressed as mean (SD) or median (IQR); independent *t*-tests were used for normally distributed values, and Mann-Whitney *U* tests were used for skewed data. Categorical variables were expressed as frequencies and percentages and analyzed using the χ2 or Fisher exact test, as appropriate. A repeated measures analysis of variance (ANOVA) was used to detect trends in score changes and treatment effects at different time points within groups. Multiple interpolation (MI) methods were used to estimate missing data for the primary and secondary outcomes. To assess the robustness of MI methods under the assumption of randomization, sensitivity analyses of the primary outcome were performed using per-protocol (PP) sets. Differences between groups were reported using 95% confidence intervals and bilateral *P* values, with values less than 0.05 considered significant.

## 3. Results

In this research, a total of 114 patients were assessed and gave their informed consent between May 2022 and November 2022. Following the exclusion of 36 patients, 78 eligible patients were enrolled and randomly assigned to two groups (39 in the MA group and 39 in the SA group).

**FIGURE 1:**
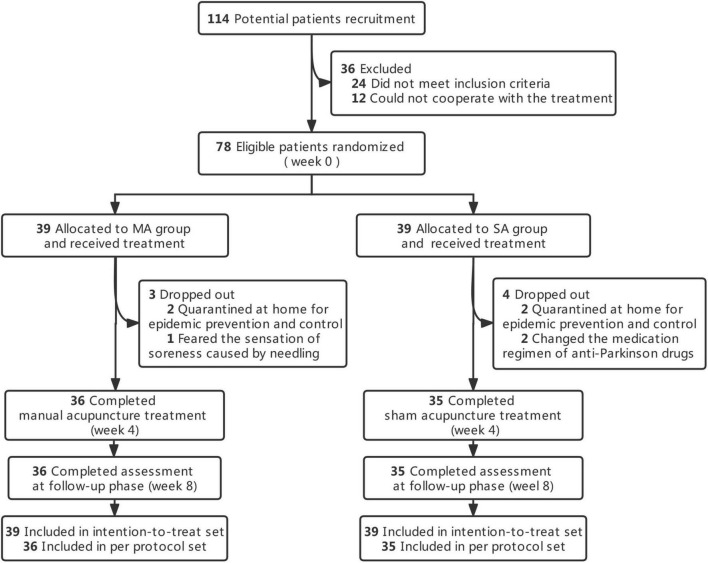
The execution and dropout details. MA, manual acupuncture; SA, sham acupuncture.

Over the course of the treatment, seven patients dropped out [dropout rate 9.0%; MA group: *n* = 3 (7.7%); SA group: *n* = 4 (10.3%)]. The cause of withdrawal was being quarantined at home for epidemic control, a sore sensation caused by a needle, or a change in the medication regimen of an anti-Parkinson’s drugs. According to the CONSORT in Figure 1, the execution and dropout details were displayed in a diagram. The features of both groups at the outset were balanced, as shown in Table 1.

**TABLE 1:** Baseline characteristics of included patients.

Characteristic	Total (*n* = 78)	MA group (*n* = 39)	SA group (*n* = 39)
Sex, No. (%)*			
Female	43 (55.10)	22 (56.41)	21 (53.85)
Male	35 (44.90)	17 (43.59)	18 (46.15)
Age, mean (SD), y*	63.82 (8.29)	63.90 (7.34)	63.74 (9.24)
Duration of PD, mean (SD), y*	5.90 (4.14)	5.74 (3.95)	6.05 (4.37)
Duration of constipation, mean (SD), y*	9.62 (7.15)	9.84 (7.35)	9.40 (7.03)
Constipation appears earlier than motor symptoms, No. (%)*			
Yes	58 (74.36)	28 (71.79)	30 (76.92)
No	20 (25.64)	11 (28.21)	9 (23.08)
Equivalent daily dose of levodopa, mean (SD), mg*	481.01 (280.29)	479.33 (286.75)	482.69 (277.43)
Weekly CSBMs (week 0), mean (SD), times*	3.23 (1.44)	3.36 (1.44)	3.10 (1.45)
CSEAS, mean (SD)*	9.45 (3.27)	9.49 (3.39)	9.41 (3.18)
CSEAS-difficulty, mean (SD)*	2.32 (0.86)	2.33 (0.87)	2.31 (0.86)
CSEAS-bristol, mean (SD)*	1.83 (1.06)	2.03 (1.01)	1.64 (1.09)
CSEAS-time, mean (SD)*	1.49 (1.18)	1.49 (1.17)	1.49 (1.21)
CSEAS-incompleteness, mean (SD)*	1.50 (1.15)	1.46 (1.14)	1.54 (1.16)
CSEAS-frequency, mean (SD)*	1.10 (1.09)	0.95 (1.05)	1.26 (1.12)
CSEAS-bloating, mean (SD)*	1.21 (1.22)	1.23 (1.31)	1.18 (1.14)
PAC-QOL, mean (SD)*	35.87 (14.29)	35.79 (13.91)	35.95 (14.83)
UPDRS, mean (SD)*	42.67 (10.40)	42.49 (11.36)	42.85 (9.50)
UPDRS I, mean (SD)*	3.41 (2.59)	3.36 (2.28)	3.46 (2.89)

*The baseline characteristics of both groups were balanced. MA, manual acupuncture; SA, sham acupuncture; PD, Parkinson’s disease; Weekly CSBMs, weekly complete spontaneous bowel movements; CSEAS, Constipation Symptom and Efficacy Assessment Scale; PAC-QOL, Patient-Assessment of Constipation Quality of Life questionnaire; UPDRS, Unified Parkinson’s Disease Rating Scale.

### 3.1. Primary outcome

**FIGURE 2:**
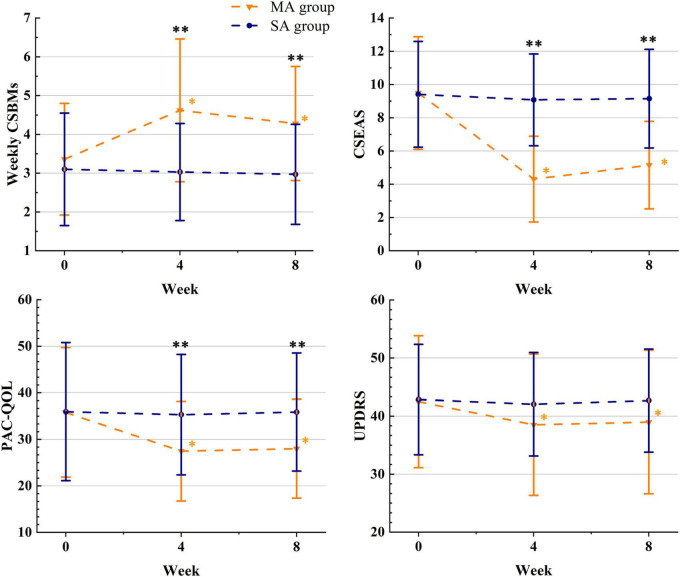
The therapeutic effects of acupuncture. *The difference with the baseline was significant within group (*P* ≤ 0.05); ^**^the difference between the groups was significant (*P* ≤ 0.05); MA, manual acupuncture; SA, sham acupuncture; Weekly CSBMs, weekly complete spontaneous bowel movements; CSEAS, Constipation Symptom and Efficacy Assessment Scale; PAC-QOL, Patient-Assessment of Constipation Quality of Life questionnaire; UPDRS, Unified Parkinson’s Disease Rating Scale.

We used ITT data; Table 2 displayed differences in outcomes between groups, while Table 3 and Figure 2 compared outcomes within groups. Weekly CSBMs in the MA group were 3.36 [standard deviation (SD), 1.44] at baseline and increased to 4.62 (SD, 1.84) after treatment (week 4). The SA group’s weekly CSBMs were 3.10 (SD, 1.45) at baseline and 3.03 (SD, 1.25) after treatment, with no significant change from baseline. Weekly CSBMs in the MA group increased from baseline by a mean of 1.26 (95% CI, 0.75 to 1.77; *P* < 0.001) at post-treatment; at follow-up, it decreased from post-treatment but still increased by 0.92 (95% CI, 0.51 to 1.34; *P* < 0.001) from baseline. Weekly CSBMs were significantly higher in the MA group compared to the SA group both after treatment and at follow-up, with a difference between groups of 1.59 (95% CI, 0.88 to 2.30; *P* < 0.001) at week 4 and 1.31 (95% CI, 0.69 to 1.93; *P* < 0.001) at week 8. We observed similar results in the PP population, as shown in Supplementary material 2 (Supplementary Table 1).

**TABLE 2:** Comparison of the primary and secondary outcomes in the manual acupuncture (MA) and sham acupuncture (SA) groups.

Outcome assessments	MA group (*n* = 39)	SA group (*n* = 39)	Difference (95% CI)	*P*
**Weekly CSBMs, mean (SD)**				
Posttreatment (week 4)	4.62 (1.84)	3.03 (1.25)	1.59 (0.88 to 2.30)	<0.001
Follow-up (week 8)	4.28 (1.47)	2.97 (1.29)	1.31 (0.69 to 1.93)	<0.001
**CSEAS, mean (SD)**				
Posttreatment	4.31 (2.58)	9.08 (2.76)	−4.77 (−5.97 to −3.57)	<0.001
Follow-up	5.15 (2.63)	9.15 (2.97)	−4.00 (−5.27 to −2.74)	<0.001
**CSEAS-difficulty, mean (SD)**				
Posttreatment	1.41 (0.88)	2.08 (1.01)	−0.67 (−1.09 to −0.24)	0.003
Follow-up	1.36 (0.84)	2.13 (0.95)	−0.77 (−1.17 to −0.36)	<0.001
**CSEAS-bristol, mean (SD)**				
Posttreatment	0.54 (0.68)	1.49 (1.02)	−0.95 (−1.34 to −0.56)	<0.001
Follow-up	0.85 (0.84)	1.67 (1.01)	−0.82 (−1.24 to −0.40)	<0.001
**CSEAS-time, mean (SD)**				
Posttreatment	0.64 (0.78)	1.54 (1.19)	−0.90 (−1.35 to −0.44)	<0.001
Follow-up	0.79 (0.89)	1.44 (1.14)	−0.64 (−1.10 to −0.18)	0.007
**CSEAS-incompleteness, mean (SD)**				
Posttreatment	0.69 (0.92)	1.54 (1.21)	−0.85 (−1.33 to −0.36)	0.001
Follow-up	0.85 (1.07)	1.36 (1.18)	−0.51 (−1.02 to −0.06)	0.048
**CSEAS-frequency, mean (SD)**				
Posttreatment	0.28 (0.56)	1.18 (0.97)	−0.90 (−1.26 to −0.54)	<0.001
Follow-up	0.59 (0.85)	1.26 (0.94)	−0.67 (−1.07 to −0.26)	0.002
**CSEAS-bloating, mean (SD)**				
Posttreatment	0.74 (0.88)	1.26 (1.19)	−0.51 (−0.98 to −0.42)	0.033
Follow-up	0.72 (0.86)	1.31 (1.20)	−0.59 (−1.06 to −0.12)	0.014
**PAC-QOL, mean (SD)**				
Posttreatment	27.46 (10.71)	35.31 (12.93)	−7.85 (−13.20 to −2.49)	0.005
Follow-up	28.00 (10.63)	35.87 (12.67)	−7.87 (−13.15 to −2.60)	0.004
**UPDRS, mean (SD)**				
Posttreatment	38.51 (12.17)	42.05 (8.91)	−3.54 (−8.35 to 1.27)	0.147
Follow-up	38.97 (12.36)	42.67 (8.87)	−3.69 (−8.54 to 1.16)	0.134
**UPDRS I, mean (SD)**				
Posttreatment	1.95 (1.83)	2.46 (1.65)	−0.51 (−1.30 to 0.27)	0.198
Follow-up	2.21 (1.56)	2.38 (1.74)	−0.18 (−0.92 to 0.57)	0.633

MA, manual acupuncture; SA, sham acupuncture; Weekly CSBMs, weekly complete spontaneous bowel movements; CSEAS, Constipation Symptom and Efficacy Assessment Scale; PAC-QOL, Patient-Assessment of Constipation Quality of Life questionnaire; UPDRS, Unified Parkinson’s Disease Rating Scale.

**TABLE 3:** Comparison of the primary and secondary outcomes from the baseline.

Variable	Mean change from baseline (95% CI)		Mean change from baseline (95% CI)	
	MA group (*n* = 39)	*P*	SA group (*n* = 39)	*P*
**Weekly CSBMs**				
Posttreatment (week 4)	1.26 (0.75 to 1.77)	<0.001	−0.08 (−0.36 to 0.21)	0.584
Follow-up (week 8)	0.92 (0.51 to 1.34)	<0.001	−0.13 (−0.42 to 0.16)	0.376
**CSEAS**				
Posttreatment	−5.18 (−6.11 to −4.25)	<0.001	−0.33 (−1.00 to 0.33)	0.318
Follow-up	−4.33 (−5.28 to −3.38)	<0.001	−0.26 (−0.88 to 0.37)	0.408
**CSEAS-difficulty**				
Posttreatment	−0.92 (−1.21 to −0.64)	<0.001	−0.23 (−0.47 to 0.10)	0.060
Follow-up	−0.97 (−1.32 to −0.63)	<0.001	−0.18 (−0.52 to 0.16)	0.292
**CSEAS-bristol**				
Posttreatment	−1.49 (−1.84 to −1.13)	<0.001	−0.15 (−0.42 to 0.11)	0.244
Follow-up	−1.18 (−1.49 to −0.87)	<0.001	0.03 (−0.18 to 0.23)	0.800
**CSEAS-time**				
Posttreatment	−0.85 (−1.15 to −0.54)	<0.001	0.05 (−0.13 to 0.23)	0.570
Follow-up	−0.69 (−1.05 to −0.33)	<0.001	−0.05 (−0.20 to 0.10)	0.487
**CSEAS-incompleteness**				
Posttreatment	−0.77 (−1.10 to −0.44)	<0.001	0.00 (−0.25 to 0.25)	1.000
Follow-up	−0.62 (−0.95 to −0.29)	0.001	−0.18 (−0.41 to 0.54)	0.128
**CSEAS-frequency**				
Posttreatment	−0.67 (−1.00 to −0.33)	<0.001	−0.08 (−0.34 to 0.19)	0.555
Follow-up	−0.36 (−0.69 to −0.03)	0.033	0.00 (−0.27 to 0.27)	1.000
**CSEAS-bloating**				
Posttreatment	−0.49 (−0.84 to −0.13)	0.009	0.08 (−0.20 to 0.35)	0.600
Follow-up	−0.51 (−0.92 to −0.11)	0.015	0.13 (−0.12 to 0.38)	0.303
**PAC-QOL**				
Posttreatment	−8.33 (−11.24 to −5.43)	<0.001	−0.64 (−2.31 to 1.02)	0.441
Follow-up	−7.80 (−10.94 to −4.65)	<0.001	−0.08 (−1.90 to 1.75)	0.932
**UPDRS**				
Posttreatment	−3.97 (−5.83 to −2.12)	<0.001	−0.80 (−2.00 to 0.41)	0.190
Follow-up	−3.51 (−5.14 to −1.89)	<0.001	−0.18 (−1.20 to 0.85)	0.725
**UPDRS I**				
Posttreatment	−1.41 (−2.12 to −0.70)	<0.001	−1.00 (−1.80 to −0.20)	<0.001
Follow-up	−1.15 (−1.77 to −0.54)	<0.001	−1.08 (−1.76 to −0.39)	<0.001

MA, manual acupuncture; SA, sham acupuncture; Weekly CSBMs, weekly complete spontaneous bowel movements; CSEAS, Constipation Symptom and Efficacy Assessment Scale; PAC-QOL, Patient-Assessment of Constipation Quality of Life questionnaire; UPDRS, Unified Parkinson’s Disease Rating Scale.

### 3.2. Secondary outcomes

Constipation symptom and efficacy assessment scale (CSEAS) was used as a secondary outcome to reflect constipation symptoms. At the end of treatment and follow-up, the decrease of CSEAS scores in the MA group was significantly greater than the decrease in the SA group (CSEAS of posttreatment: between-group difference, −4.77) (95% CI, −5.97 to −3.57; *P* < 0.001); (CSEAS of follow-up: between-group difference, −4.00) (95% CI, −5.27 to −2.74; *P* < 0.001). We compared the symptoms of the six aspects of CSEAS in patients with PDC and discovered that acupuncture was more effective in improving defecation straining (Difficulty), stool properties (Bristol), and prolonged defecation (Time), as shown in Figure 2.

The PAC-QOL and the UPDRS were used to assess patients’ quality of life and overall condition with PD. In terms of life improvement, the PAC-QOL score of the MA group was significantly lower than that of the SA group after treatment and at follow-up, with significant differences between groups (PAC-QOL of posttreatment: between-group difference, −7.85) (95% CI, −13.20 to −2.49; *P* = 0.005); (PAC-QOL of follow-up: between-group difference, −7.87) (95% CI, −13.15 to −2.60; *P* = 0.004). In terms of overall condition with PD, we discovered that the comparison of UPDRS scores between the MA and SA groups was not significantly different at both week 4 and week 8 (UPDRS of posttreatment: between-group difference, −3.54) (95% CI, −8.35 to 1.27; *P* = 0.147); (UPDRS of follow-up: between-group difference, −3.69) (95% CI, −8.54 to 1.16; *P* = 0.134). However, there was a significant difference in UPDRS scores in the MA group after treatment and at follow-up compared to baseline, with a change of −3.97 (95% CI, −5.83 to −2.12; *P* < 0.001) at post-treatment and −3.51 (95% CI, −5.14 to −1.89; *P* < 0.001) at follow-up.

### 3.3. Evaluation of safety and blinding

During the trial, six patients (7.6%) in the MA group experienced at least one adverse event, while none in the SA group did. There were no serious adverse events in either group. Supplementary Table 2 of the Supplementary material 2 depicted details of adverse events. When assessing the effectiveness of the blinded treatment at the end of the study, we discovered no discernible difference in patients’ ability to correctly guess the assignment status between the MA and SA groups (*p* = 0.276). Blinding assessments is depicted in Supplementary Table 3 of Supplementary material 2.

## 4. Discussion

To our knowledge, this is the first randomized controlled trial to evaluate the efficacy of acupuncture for the treatment of PDC. We found that manual acupuncture was more effective than sham acupuncture at alleviating constipation symptoms and enhancing quality of life in PD patients after a 4-week course of treatment. The therapeutic effect was sustained throughout the follow-up.

Prolonged colonic transit time was described as one of the main mechanisms of PDC (Zhang et al., 2021; Safarpour et al., 2022). It has been reported that acupuncture can promote gastrointestinal motility by stimulating the distal colon through parasympathetic activation (Li et al., 2007). Weekly CSBMs were the most direct tool for evaluating colon transit in constipation. Previous research showed that manual acupuncture improves weekly CSBMs in patients with chronic constipation more effectively than sham acupuncture (Zhang et al., 2020). The PDC patients in our study had similar positive outcomes. The minimal clinically important difference (MCID) in weekly CSBMs is an increase of 1 unit, which is considered clinically significant for the relief of constipation (Liu et al., 2016). In this study, the change from baseline in weekly CSBMs in the MA group was 1.26 (95% CI, 0.75 to 1.77) after treatment, meeting the MCID. However, at follow-up, the increase from baseline in weekly CSBMs in the MA group was 0.92 (95% CI, 0.75 to 1.77), which did not meet the MCID. That means that during follow-up, although there was a statistically significant increase in CSMBs in the MA group, there was not a clinically significant difference. It is inconsistent with previous studies in functional constipation (Liu et al., 2016), which indicated that there were clinically significant differences during follow-up. This difference shows that due to the complex pathogenesis of PDC, it is more likely to recur than common functional constipation, which requires extended cycles of treatment to maintain a long-term therapeutic effect.

A study investigating the frequency of gastrointestinal symptoms in patients with PD showed that they were consistently plagued by defecation straining, hard stools, prolonged defecation, the sensation of incomplete evacuation, and bloating (Sung et al., 2014). In this study, we used the CSEAS with six different dimensions to measure the improvement of these symptoms in PDC. The results showed that patients in the MA group had better improvement in the above-mentioned gastrointestinal symptoms than those in the SA group during both the treatment and follow-up periods. Previous clinical trials reported similar results of acupuncture in treating hard bowel movements and hard stools (Xu et al., 2020), though few studies evaluated prolonged defecation as well as the sense of incomplete evacuation and bloating. The severe and frequent gastrointestinal symptoms were positively correlated with the severe motor symptoms (Sun et al., 2021). Patients with bradykinesia, tremor, and hypertonia found it difficult to stay in the bathroom for long periods of time. Additionally, patients who experienced bloating and the feeling of incomplete evacuation had a diminished appetite and had to be careful about what they ate. In this study, shorter defecation times and reduced symptoms of incomplete evacuation and bloating were seen in the MA group compared to the SA group during both treatment and follow-up. These findings offered more proof that acupuncture can effectively relieve constipation in PD.

Enhancing the quality of life in relation to health is one of PDC treatment’s objectives (Sauerbier et al., 2017). In this study, the PAC-QOL, which included the dimensions of psychosocial discomfort, physical discomfort, satisfaction, and worriedness and concerns, was used to assess the quality of life in patients with PDC. We conducted the PAC-QOL in a Chinese version, which has proven reliable and valid (Zhao, 2011). The change in PAC-QOL scores revealed that MA significantly improved the quality of life of PDC patients after treatment when compared to SA. Both results during treatment and follow-up in the MA group were greater than the MCID (at least a 0.5-point reduction in the total PAC-QOL score) (Liu et al., 2016), supporting the clinical effectiveness of acupuncture in improving quality of life in PDC patients.

As is well-known, PD is a progressive neurodegenerative disorder, and there is no treatment available to slow disease progression (Bloem et al., 2021). In our study, when assessing overall PD symptoms, the UPDRS scores were reduced in both the MA and SA groups, but there was no difference between the two treatment groups. Further research found that the decrease in scores was primarily concentrated in UPDRS I (the mental, behavioral, and emotional components). Previous studies have shown that placebo effects are usually observed when acupuncture is used for improving psychiatric and psychological-related symptoms in patients with functional gastrointestinal disorders as well as Parkinson’s disease (Kaptchuk, 2020; Li et al., 2022; Fan et al., 2022b). Therefore, we can infer that acupuncture was helpful in improving mental and psychological symptoms in PDC patients, even though it may have been a placebo effect.

Overall, acupuncture can effectively alleviate constipation symptoms and improve the quality of life for PDC patients.

There are some strengths and limitations to this study. First, a well-designed sham acupuncture device demonstrated the reliability of the difference between manual and sham acupuncture. It maintained the sensation of the needles touching the skin without piercing it, which ensured physiologically inert blindness. Patients in both groups did not differ significantly in their ability to correctly guess their distribution at the end of the study, confirming that the blinding was psychologically credible. Secondly, we used the CSEAS, a well-designed scale, to measure the improvement of the constipation symptom. It was designed in 2015 by the Anorectal Surgery Group of the Chinese Medical Association’s Surgery Branch. The CSEAS comprised six questions covering six constipation-related symptoms and was suitable for Chinese patients. Its succinct question reflected the efficiency of the treatment in a comprehensive and targeted way. In this study, we analyzed each symptom to provide more detail on the efficacy of acupuncture in improving PDC. Thirdly, no serious adverse events were found in the study, and the few adverse events that occurred in the MA group were mild and self-resolving, like those reported in previous acupuncture studies. This provided assurance of the safety of acupuncture for PDC.

In the later stages of PD, the short- and long-term responses to dopaminergic drugs are reduced, and the inability to store excess dopamine necessitates higher and more frequent doses of levodopa (Rosqvist et al., 2018). Some studies suggested that improving constipation might help regulate the absorption of dopaminergic drugs in the intestine, thereby delaying this phenomenon (Güneş and Karavana, 2022). A limitation of this study is that the study period was too short, and we were unable to observe the effect of acupuncture on levodopa dose by improving constipation. In order to better understand the association between improvement of bowel movements and levodopa dosage following acupuncture treatment, the follow-up period should be extended, and prospective cohort studies should be developed. Another limitation is the absence of a Chinese version of The Gastrointestinal Dysfunction Scale for Parkinson’s disease, which might bias our evaluation of PDC patients. In the future, we will work on a Chinese version of this scale for evaluating patients with PDC. Furthermore, this study lacked objective markers that approached mechanisms. The following step will be to incorporate objective markers to advance our understanding of how acupuncture treats PDC.

## 5. Conclusion

Acupuncture was discovered to effectively relieve constipation symptoms and improve the quality of life of PD patients, with the treatment effect lasting up to 4 weeks.

## Data availability statement

The raw data supporting the conclusions of this article will be made available by the authors, without undue reservation.

## Ethics statement

The studies involving human participants were reviewed and approved by the Ethics Committee of the First Affiliated Hospital of Guangzhou University of Chinese Medicine (Ethics number: K-2022-005). The patients/participants provided their written informed consent to participate in this study.

## Author contributions

Y-JL, J-QF, and L-XZ conceived and designed the experiments. L-XZ assessed the potential patients and supervised the acupuncture scheme. M-YY, XL, W-JL, and Y-YC performed the treatment. I-IL and J-QF assessed the outcomes. Y-JL analyzed the data. W-QT generated the random allocation and assigned the participants. Y-JL, I-IL, and J-QF wrote the manuscript. Y-TW designed the acupuncture device. All authors approved the final manuscript.
