# Supplementary material for: Efficacy of acupuncture for the treatment of Parkinson’s disease-related constipation (PDC): A randomized controlled trial
Source: Front Neurosci. 2023 Feb 13;17:1126080. doi: 10.3389/fnins.2023.1126080 (PMC9972583; doi:10.3389/fnins.2023.1126080)
Supplement: Supplementary file 1 [file Data_Sheet_1.docx]

**Supplementary Material 1**

**Protocol of trial**

**Table of Contents**

[1. General informational 2](#_Toc126432290)

[1.1 Institution 2](#_Toc126432291)

[1.2 Investigators 2](#_Toc126432292)

[2.Study Design 2](#_Toc126432293)

[2.1 Study Overview 2](#_Toc126432294)

[2.2 Background 2](#_Toc126432295)

[2.3 Study Objectives 3](#_Toc126432296)

[2.4 Methodology 3](#_Toc126432297)

[2.4.1 Trial design 3](#_Toc126432298)

[2.4.1.1 Pilot study and Sample size 3](#_Toc126432299)

[2.4.1.2 Randomization 4](#_Toc126432300)

[2.4.1.3 Blinding 4](#_Toc126432301)

[2.4.2 Diagnosis criteria 6](#_Toc126432302)

[2.4.2.1Diagnostic criteria for Parkinson's disease 6](#_Toc126432303)

[2.4.2.2 Diagnostic criteria for functional constipation 7](#_Toc126432304)

[2.4.3 Subjects 7](#_Toc126432305)

[2.4.3.1 Inclusion criteria 7](#_Toc126432306)

[2.4.3.2 Exclusion criteria 8](#_Toc126432307)

[2.4.3.3 Shedding and rejection criteria 8](#_Toc126432308)

[2.4.4 Trial flow Chart 8](#_Toc126432309)

[2.4.5 Outcomes Measurements 10](#_Toc126432310)

[2.4.5.1 Primary Outcome 10](#_Toc126432311)

[2.4.5.2 Secondary Outcomes 10](#_Toc126432312)

[2.4.5.3 Evaluation of blinding effects 11](#_Toc126432313)

[3. Safety Assessment 11](#_Toc126432314)

[4. Interventions 11](#_Toc126432315)

[4.1 Basic treatment of Parkinson's disease 11](#_Toc126432316)

[4.2 Treatment (Manual acupuncture group) 11](#_Toc126432317)

[4.2.1 Acupuncture preparation 11](#_Toc126432318)

[4.2.2 Acupoints selection 12](#_Toc126432319)

[4.2.3 Operation method 12](#_Toc126432320)

[4.3 Control group (Sham acupuncture group) 13](#_Toc126432321)

[4.3.1 Acupuncture preparation 13](#_Toc126432322)

[4.3.2 Acupoints selection 13](#_Toc126432323)

[4.3.3 Operation method 13](#_Toc126432324)

[4.4 Emergency treatment 13](#_Toc126432325)

[5. Statistical analysis and processing 14](#_Toc126432326)

[6. Quality Control 14](#_Toc126432327)

[7. Ethical Approval 14](#_Toc126432328)

[8. Informed Consent 14](#_Toc126432329)

[9. References 19](#_Toc126432330)

1. General informational

1.1 Institution

Outpatient department of Parkinson's disease, the First Affiliated Hospital of Guangzhou University of Chinese Medicine.

1.2 Investigators

Li-Xing Zhuang, Professor, Designer and supervisor of acupuncture scheme, enrolled participants

Ying-Jia Li, MM, Designer of acupuncture scheme, Data statistics and sorting

IAN-I LEONG, MM, Outcome evaluation, Quality Control.

Jing-Qi Fan, PhD, Designer of acupuncture scheme, Outcome evaluation

Ming-Yue Yan, MM, Implementation of experiment

Xin Liu, PhD, Implementation of experiment

Wei-Jing Lu, MM, Implementation of experiment

Yuan-Yuan Chen, MM, Implementation of experiment

Yu-Ting Wang, PhD, Designer of the acupuncture device

Wei-Qiang Tan, MM, Generated the random allocation and assigned participants

2.Study Design

2.1 Study Overview

The overview of this study was to assess the clinical efficacy of acupuncture therapy in the treatment of Parkinson's disease-related constipation (PDC).

2.2 Background

Constipation is a non-motor symptom of Parkinson's disease (PD)(Bloem et al., 2021). More than 50% of PD patients experience constipation, which is an autonomic dysfunction characterized by anorectal disturbances and delayed colonic transit(Gan et al., 2018). Current research indicates that some PD patients experience constipation years before motor symptoms(Lesser, 2002). With the progression of PD, the prevalence of constipation increases, negatively impacting patients' quality of life. Constipation is developing into a significant problem in parallel with motor symptoms(Schapira et al., 2017).

Currently, increasing dietary fiber and fluid intake is commonly used to treat PDC(Barboza et al., 2015). When lifestyle changes are unhelpful, probiotics and prebiotic fiber, macrogol, and lubiprostone may be used, though these have limited evidence for treating PDC(Mozaffari et al., 2020). Constipation was recurrent and refractory in most PD patients, which requires maintenance therapy. As PD progresses, patients must switch to higher doses of anti-Parkinson's drugs to improve motor symptoms(Chou et al., 2018). Treating constipation with non-pharmacologic therapies is more appealing to patients with PD.

Acupuncture is a popular non-pharmacological treatment for gastrointestinal and neurological conditions, and it has been shown to be effective in treating constipation and PD in several studies(Li et al., 2022). However, high-quality clinical evidence on the efficacy of acupuncture for PDC is lacking. As a result, we created a randomized, single-blind clinical trial with sham acupuncture (SA) as a control to evaluate the efficacy of manual acupuncture (MA) for PDC.

2.3 Study Objectives

We designed a randomized controlled clinical trial to compare the efficacy and safety of manual acupuncture (MA) to sham acupuncture (SA) in the treatment of PDC.

2.4 Methodology

2.4.1 Trial design

This is a randomized, sham-controlled clinical trial aiming to examine the effect of acupuncture in the treatment of PDC. The cases in this study will be recruited from patients with PDC who meet the inclusion criteria in the outpatient department of Parkinson's disease at the First Affiliated Hospital of Guangzhou University of Chinese Medicine between May 2022 and November 2022. Participants will be fully informed of the study purpose, procedure, and related rights before the start of this study and will have signed an informed consent form to protect their rights, safety, and privacy.

The clinical trial has been approved by the Ethics Committee of the First Affiliated Hospital of Guangzhou University of Chinese Medicine (Ethics number: K-2022-005). The protocol has been registered with the China Clinical Trials Center (ChiCTR2200059979).

2.4.1.1 Pilot study and Sample size

We conducted a pilot study to determine the sample size for the randomized controlled trial. Twenty-eight patients were chosen to receive either manual or sham acupuncture (14 patients in each group). We used the change in weekly complete spontaneous bowel movements (CSBMs) from baseline to the end of the trial as an evaluation. Patients who received MA had a mean CSBMs score of 0.964 (standard deviation [SD],1.726), whereas patients who received SA had a score of -0.321. (SD, 1.310). According to PASS version 15.0, 31 patients are needed for each group at a two-sided significance level of 5% and a power of 90%. The sample size estimation was shown in Supplementary Figure S1. With an estimated loss-to-follow-up rate of 20%, we will plan to enroll a total of 78 patients with 39 patients per group.


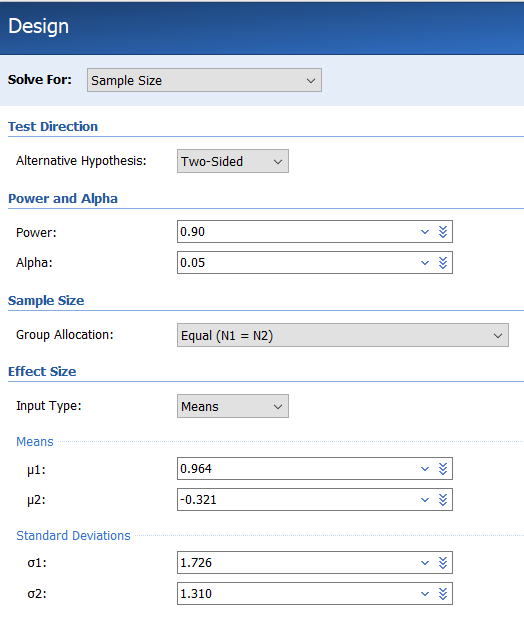


**Supplementary Figure S1 Sample size estimation**

2.4.1.2 Randomization

An independent researcher will use the SPSS 26.0 procedure to generate the random number tables. 78 patients will be randomly assigned to the MA group and the SA group in a ratio of 1:1. The information about the assigned groups will be made into cards with the same shape and placed in opaque envelopes. When the patients start the clinical study, the envelopes will be opened and the groups will be formed according to the instructions on the randomized cards.

2.4.1.3 Blinding

This study will be blinded to patients, outcome assessors, and statisticians. The treatment time for patients in different groups will be set at different times of the day to prevent communication between the two groups. During the treatment, the patients will be required to wear an eye mask to ensure the implementation of the blinding method. All operations will be performed according to a standardized procedure to ensure consistency of operation. The efficacy assessor will not be involved in the grouping and treatment manipulation. The blinding will be removed at the end of the treatment, and patients will be informed of their grouping after treatment as well as will be told their type of acupuncture after follow-up.

This study will be controlled using a special acupuncture device which was developed and designed by our team members. The special acupuncture device was shown in Supplementary Figure S2. The acupuncture device consisted of a base, a cannula, and a specially designed acupuncture needle. There are classified into two types of bases. In the MA group, the base has a longitudinal opening at the bottom, through which acupuncture needles can be pierced into the skin. Conversely, the SA group's base has a closed adhesive with no opening at the bottom, so special flat-head needling can contact the skin through the adhesive but cannot pierce it. The cannula is removable and can be adjusted to meet the needs of various needling angles depending on the site of needling. The special flat-head needle has the same shank as ordinary acupuncture needle. Since the flat-head needle do not actually pierce the skin, it is designed to be shorter in length than ordinary acupuncture needle, and the length of the body exposed during needling is the same as that of ordinary acupuncture needle to ensure consistency of appearance during operation.

The special acupuncture device to be used in this study has been approved for a national utility model patent, with the patent number ZL202121352221.7 and is produced by a manufacturer with relevant qualifications (Guangzhou Suixin Medical Equipment Co., Ltd.). The related article has been published(Wang et al., 2022).


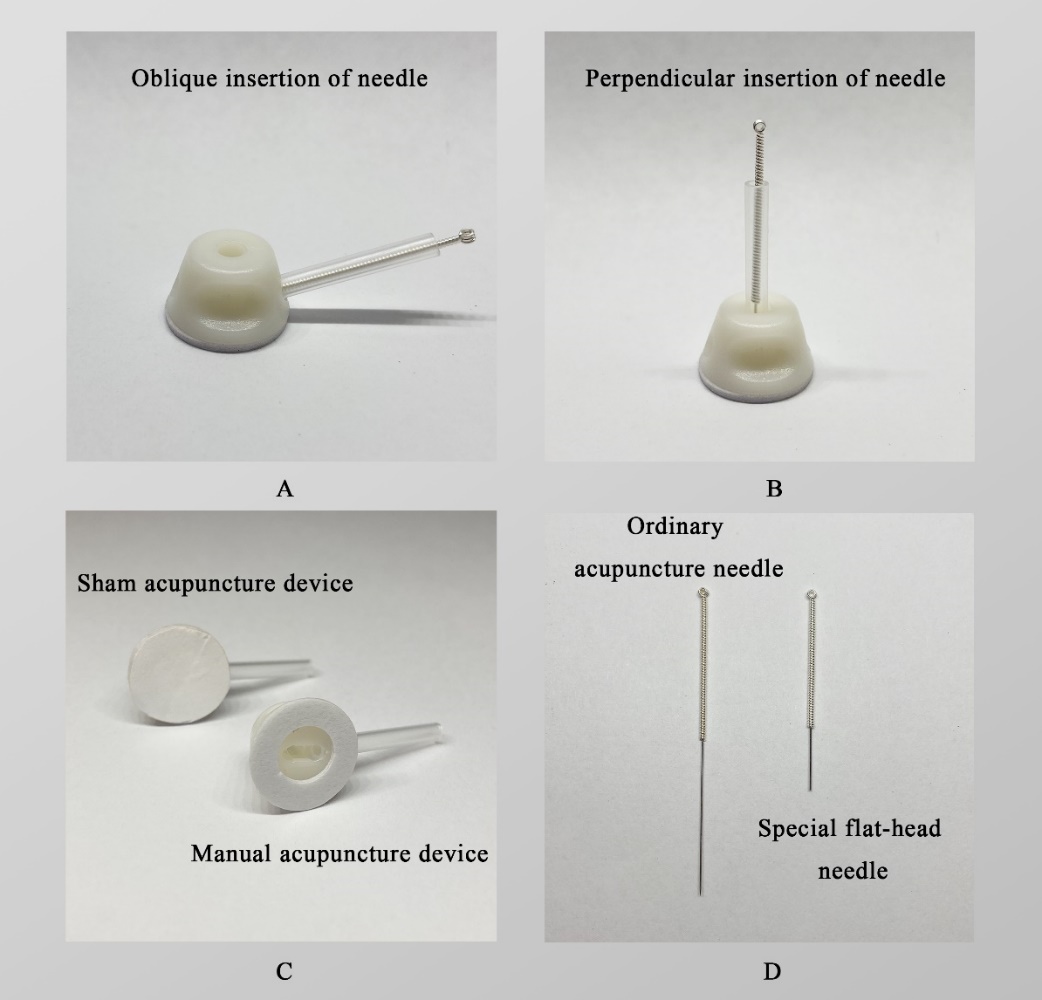


**Supplementary Figure S2 Details of the special device, acupuncture needles, and the angle of needle insertion.**

2.4.2 Diagnosis criteria

2.4.2.1Diagnostic criteria for Parkinson's disease

The diagnostic criteria for PD will refer to the Movement Disorder Society's revised clinical diagnostic criteria for Parkinson's disease in 2015(Postuma et al., 2015), which includes: there should be at least two supporting criteria, no absolute exclusion criteria, and no red flags. If one red flag is present, there must also be at least one supportive criterion and no more than 2 red flags are allowed for this category.

**2.4.2.1.1Supportive criteria**

(1) Clear and dramatic beneficial response to dopaminergic therapy. During initial treatment, patient returned to normal or near-normal level of function.

(2) Presence of levodopa-induced dyskinesia.

(3) Rest tremor of a limb, documented on clinical examination (in past, or on current examination).

**2.4.2.1.2 Absolute exclusion criteria**

(1) Unequivocal cerebellar abnormalities, such as cerebellar gait, limb ataxia, or cerebellar oculomotor abnormalities.

(2) Downward vertical supranuclear gaze palsy, or selective slowing of downward vertical saccades.

(3) Diagnosis of probable behavioral variant frontotemporal dementia or primary progressive aphasia, defined according to consensus criteria within the

first 5 years of disease.

(4) Parkinsonian features restricted to the lower limbs for more than 3 years.

(5) Treatment with a dopamine receptor blocker or a dopamine-depleting agent in a dose and time-course consistent with drug-induced parkinsonism.

(6) Absence of observable response to high-dose levodopa despite at least moderate severity of disease.

(7) Unequivocal cortical sensory loss, clear limb ideomotor apraxia, or progressive aphasia.

(8) Normal functional neuroimaging of the presynaptic dopaminergic system.

(9) Documentation of an alternative condition known to produce parkinsonism and plausibly connected to the patient’s symptoms, or, the expert evaluating physician, based on the full diagnostic assessment feels that an alternative syndrome is more likely than PD.

**2.4.2.1.3 Red flags**

(1) Rapid progression of gait impairment requiring regular use of wheelchair within 5 years of onset.

(2) A complete absence of progression of motor symptoms or signs over 5 or more years unless stability is related to treatment.

(3) Early bulbar dysfunction: severe dysphonia or dysarthria (speech unintelligible most of the time) or severe dysphagia (requiring soft food, NG tube, or gastrostomy feeding) within first 5 years.

(4) Inspiratory respiratory dysfunction: either diurnal or nocturnal inspiratory stridor or frequent inspiratory sighs.

(5) Severe autonomic failure in the first 5 years of disease. This can include: Orthostatic hypotension, Severe urinary retention, or urinary incontinence.

(6) Recurrent (>1/years) falls because of impaired balance within 3 years of onset.

(7) Disproportionate anterocollis (dystonic) or contractures of hand or feet within the first 10 years.

(8) Absence of any of the common nonmotor features of disease despite 5 years disease duration. These include sleep dysfunction (sleep-maintenance insomnia, excessive daytime somnolence, symptoms of REM sleep behavior disorder), autonomic dysfunction (constipation, daytime urinary urgency, symptomatic orthostasis), hyposmia, or psychiatric dysfunction (depression, anxiety, or hallucinations).

(9) Otherwise-unexplained pyramidal tract signs, defined as pyramidal weakness or clear pathologic hyperreflexia (excluding mild reflex asymmetry and isolated extensor plantar response).

(10) Bilateral symmetric parkinsonism. The patient or caregiver reports bilateral symptom onset with no side predominance, and no side predominance is observed on objective examination.

2.4.2.2 Diagnostic criteria for functional constipation

The Rome IV diagnostic criteria for functional constipation which published by the American Rome Committee in 2016 will be selected for the clinical diagnosis of functional constipation(Palsson et al., 2016).

(1) Two or more of the following must be met (a-e must be met at least once in every 4 bowel movements): (a) Straining to defecate. (b) Lumpy or hard bowel movements. (c) A feeling of incomplete defecation. (d) A sense of anorectal obstruction and/or obstruction. (e) Requires manual manipulation (e.g., finger-assisted defecation, pelvic floor support for defecation) to facilitate defecation. (f) Defecation less than 3 times per week.

1. Almost no loose stools without laxatives.
2. Inadequate conditions for the diagnosis of IBS.
3. Symptoms have been present for at least 6 months prior to diagnosis, and the above criteria have been met for the last 3 months.

2.4.3 Subjects

2.4.3.1 Inclusion criteria

(1) Meeting the Movement Disorder Society's revised clinical diagnostic criteria for Parkinson's disease in 2015.

(2) Meeting the diagnostic criteria for functional constipation in the Rome IV diagnostic Criteria.

(3) Between 35 and 80 years of age.

(4) Those with Hoehn-Yahr grade of Parkinson's disease ≤ 3.

(5) No medications taken within 2 weeks prior to treatment that may affect gastrointestinal function, such as prucalopride and probiotics.

(6) No participation in other clinical trials such as drugs or acupuncture within 1 month prior to inclusion in this study.

(7) Voluntary engagement in this study and ability to sign the informed consent.

(8) Good compliance, ability to understand the study content, and cooperation in the completion of the bowel symptom diary and scale filling.

2.4.3.2 Exclusion criteria

1. Noncompliance with inclusion criteria.
2. Having organic lesions of the digestive system (such as intestinal adhesions, obstruction, tumors or malformations in the gastrointestinal tract, etc.), or a history of abdominal or anorectal surgery that may affect intestinal transit, or systemic diseases that affect the dynamics of the digestive tract (such as diabetes mellitus, hyperthyroidism, etc.).
3. Serious life-threatening diseases such as severe cardiovascular and malignant tumors, etc.
4. Those who have skin lesions that are not inappropriate for needling, or those who have viscose allergy that prevent acupuncture device attachment.
5. Pregnant or lactating women.

2.4.3.3 Shedding and rejection criteria

A case is considered to be shed if any of the following criteria are met.

(1) Refusal to cooperate with the study arrangements for personal reasons and no longer suitable to continue the study after assessment.

(2) Sudden onset of serious illness, who is assessed to be unfit to continue with the study.

(3) The occurrence of a serious adverse event or an unexpected event.

(4) Those who withdraw from the study on their own.

A case is considered to be rejected if the following conditions are met.

(1) Those who have not completed treatment as required by this study, or the dose or dosing regimen of the anti-Parkinsonian drug need to be adjusted during treatment.

2.4.4 Trial flow Chart

The trial flow chart and the schedule of enrollment, interventions, and assessments are shown in Supplementary Figure S3 and Table S1.


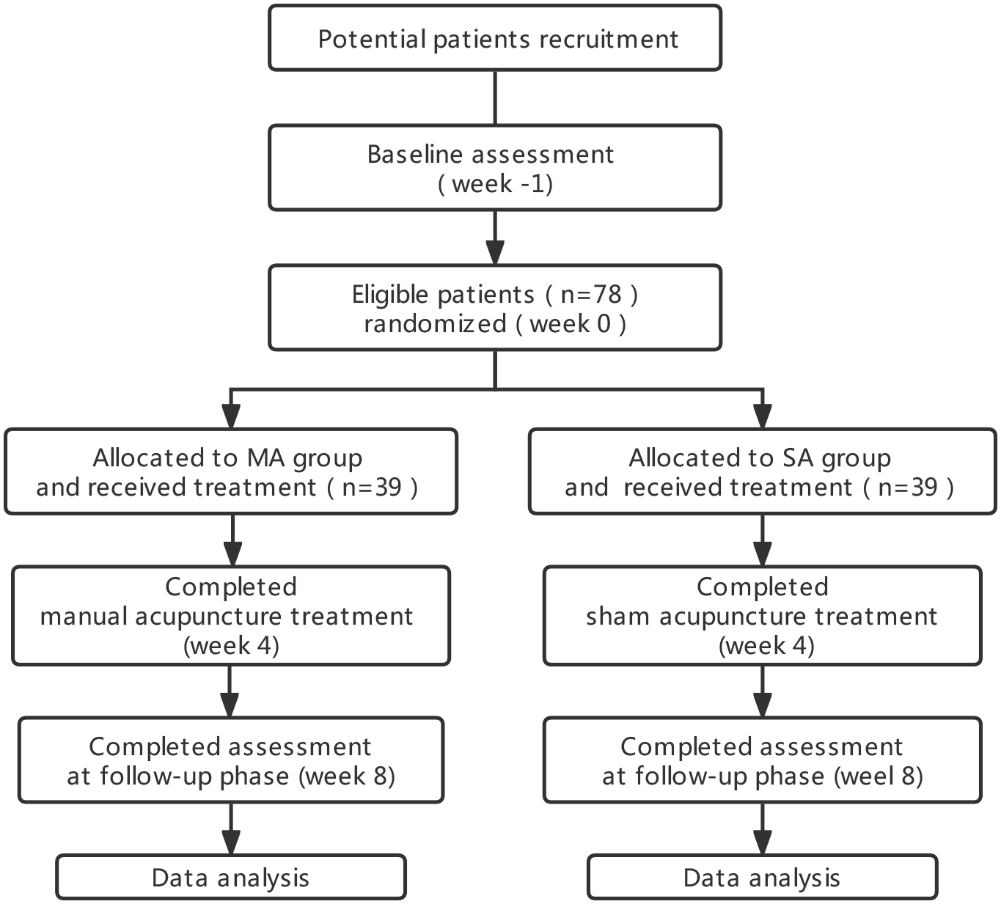


**Supplementary Figure S3. Trial flow chart**

**Table S1. Time of visit and data collection**

|  | **Baseline** | **Allocation** | **Treatment** | | | | | **Follow-up** | |
| --- | --- | --- | --- | --- | --- | --- | --- | --- | --- |
| Time point (Week) | -1 | 0 | 1 | 2 | | 3 | 4 | 8 |  |
| **Enrollment** |  | | | |  |  |  |  |  |
| Signing consent | **×** |  |  |  | |  |  |  |  |
| Screening | **×** |  |  |  | |  |  |  |  |
| Dose of levodopa | **×** |  |  |  | |  |  |  |  |
| Randomization |  | **×** |  |  | |  |  |  |  |
| **Interventions** |  | | | |  |  |  |  |  |
| Manual acupuncture group |  |  | **×** | **×** | | **×** | **×** |  |  |
| Sham acupuncture group |  |  | **×** | **×** | | **×** | **×** |  |  |
| **Assessments** |  | | | |  |  |  |  |  |
| Weekly CSBMs | **×** | **×** | **×** | **×** | | **×** | **×** | **×** |  |
| CSEAS |  | **×** |  |  | |  | **×** | **×** |  |
| PAC-QOL |  | **×** |  |  | |  | **×** | **×** |  |
| UPDRS |  | **×** |  |  | |  | **×** | **×** |  |
| Assessment of blinding |  | **×** |  |  | |  |  | **×** |  |
| Adverse events |  |  | **×** | **×** | | **×** | **×** |  |  |

Weekly CSBMs= weekly complete spontaneous bowel movements; CSEAS=Constipation Symptom and Efficacy Assessment Scale; PAC-QOL= Patient-Assessment of Constipation Quality of Life questionnaire; UPDRS= Unified Parkinson’s Disease Rating Scale.

2.4.5 Outcomes Measurements

2.4.5.1 Primary Outcome

**2.4.5.1.1** **The change of weekly complete spontaneous bowel movements (CSBMs).**

We use weekly CSBMs to observe changes in the number of bowel movements in patients with constipation. CSBMs are defined as the number of bowel movements that have occurred in the past 24 hours without the use of emergency medication. CSBMs are recorded by patients on an electronic bowel diary under the direction of an independent outcome assessor. Weekly CSBMs will be collected at baseline (week 0), post-treatment (week 4), and follow-up (week 8), and differences from baseline levels are compared at weeks 4 and 8.

2.4.5.2 Secondary Outcomes

**2.4.5.2.1Constipation Symptom and Efficacy Assessment Scale (CSEAS)**

The Constipation Symptom and Efficacy Assessment Scale (CSEAS) is used to assess the improvement of the patient's symptoms and the efficacy of treatments in patients with constipation. It consists of six dimensions, including Difficulty, Bristol, Time, Incompleteness, Frequency, and Bloating. The score for each dimension ranges from 0 to 3 points. The total score ranges from 0 to 18, with higher scores indicating more severe constipation. Table S2 depicts details of the CSEAS. The CSEAS will be evaluated at baseline, post-treatment, and at follow-up.

**Table S2. Details of the Constipation Symptom and Efficacy Assessment Scale (CSEAS)**

| Score | Difficulty: painful evacuation effort | Bristol:  the stool properties criteria | Time: munities in lavatory per attempt (min) | Completeness: feeling incomplete evacuation | Frequency (days/time) | Bloating: abdominal distention |
| --- | --- | --- | --- | --- | --- | --- |
| 0 | Never | 7~4 | ＜10 | Never | 1~2 | Never |
| 1 | Rarely | 3 | 10~15 | Rarely | 3 | Rarely |
| 2 | Sometimes | 2 | 15~25 | Sometimes | 4~5 | Sometimes |
| 3 | Always | 1 | ＞25 | Always | ＞5 | Always |

**2.4.5.2.2 Patient-Assessment of Constipation Quality of Life (PAC-QOL)**

The PAC-QOL is used to assess the quality of life of patients with constipation and consists of 28 items with 4 dimensions: physical discomfort, psychosocial discomfort, satisfaction, and worriedness and concerns. Each item's score ranges from 0 to 4. The total score ranges from 0 to 112, with lower scores indicating better quality of life in patients with constipation. The PAC-QOL will be rated at baseline, post-treatment, and follow-up.

**2.4.5.2.3 Unified Parkinson's Disease Rating Scale (UPDRS)**

The UPDRS is a common scale used to assess the general condition of PD patients, mainly focusing on motor symptoms, activities of daily living ability, degree of disease progression, post-treatment status, side effects of treatment and complications of PD patients. The UPDRS consists of four dimensions: the UPDRS I is used to assess the mental, behavioral, and emotional symptoms; the UPDRS II is used to assess the ability of daily living; the UPDRS III is used to assess the motor signs of PD patients; and the UPDRS IV is used to assess the comorbidity. The UPDRS will be assessed at baseline, post-treatment, and follow-up.

2.4.5.3 Evaluation of blinding effects

At the end of the trial, we will ask patients to guess their assignment status and whether they think the acupuncture needle entered the skin in order to evaluate the impact of blinding.

3. Safety Assessment

The vital signs of the patients will be recorded before and after the treatment, and the occurrence of acupuncture-related adverse effects, such as needle dizziness, needle bending, needle stagnation, needle breakage, bleeding, and subcutaneous hematoma, will be described in detail during the treatment. In the event of any adverse event caused by acupuncture, it will be promptly treated, and recorded in the "Adverse Event Record Form" of the case observation form. In the event of serious adverse events, acupuncture treatment will be discontinued immediately, and the record and treatment will be in accordance with the standards and reported to the Ethics Committee of the First Affiliated Hospital of Guangzhou University of Chinese Medicine. After obtaining the patient's wish, the physician will determine whether the patient is still suitable for study participation.

4. Interventions

4.1 Basic treatment of Parkinson's disease

Both groups will be given basic treatment for PD. Throughout the study, patients in both the MA and SA groups will be required to maintain effective therapeutic doses of anti-Parkinson's drugs at levels like their baseline. Patients with any dosage adjustment, whether an increase, decrease, or adjustment, will be dropped.

4.2 Treatment (Manual acupuncture group)

4.2.1 Acupuncture preparation

(1) Disposable, sterile, acupuncture needles (0.30 × 25 mm, 0.30 × 40 mm, Suzhou Tianxie Medical Supplies Co., Ltd., Suzhou, China); (2) Acupuncture device with a longitudinal opening at the bottom (Guangzhou Suixin Medical Supplies Co., Ltd., Guangzhou, China).

4.2.2 Acupoints selection

All acupoints are selected based on traditional Chinese medicine theory and previous articles on PD and constipation(Li et al., 2019; Nazarova et al., 2022), and they are taken bilaterally and positioned in accordance with the National Standard of the People's Republic of China (GB/T12346-2006).

Acupoints: Sishenzhen (four acupoints, consisting of GV 21, GV19, and next to GV20 1.5 cun bilateral), GV24 (Shenting), GV29 (Yintang), ST25 (Tianshu), CV4 (Guanyuan), and ST37 (Shangjuxu).

4.2.3 Operation method

The operation method will be referred to in the textbook Acupuncture and Moxibustion, edited by Wang Fuchun in 2021. The depth of acupuncture will be referred to in the textbook Meridian and Acupoints, edited by Shen Xueyong in 2021.

The patients will be placed in a supine position and wear an eye patch. The physicians will precisely locate the acupoints according to the standard, routinely disinfect the skin of the patients, paste the acupuncture device, adjust the cannula according to the application site, and take the needles to puncture the skin quickly and painlessly.

Table S3 displays acupoints with their insertion depth and direction. All acupoints will be left in place for 30 minutes before being removed, and the needle holes will be pressed for a few moments with a sterilized dry cotton swab when the needles are out. All patients will receive 12 sessions of treatment, three times a week (Tuesday, Thursday, and Saturday), for a period of four weeks.

**Table S3 Acupoints details**

| **Acupoints** | **Location** | **Direction and depth of insertion** |
| --- | --- | --- |
| Sishenzhen  (Four acupoints, consisting of GV21, GV19, and next to GV20 1.5 cun bilateral) | Four points at the vertex of the scalp, grouped around DU20 and located 1.5 cun anterior, posterior, and lateral to it. | Oblique/  0.5 to 0.8 cun |
| Shenting (GV24) | 0.5 cun directly above the midpoint of the anterior hairline. | Oblique/  0.5 to 0.8 cun |
| Yintang (GV29) | On the forehead, at the midpoint between the two medial ends of the eyebrow. | Oblique/  0.5 to 0.8 cun |
| Tianshu (ST25) | 2 cun lateral to the centre of the umbilicus. | Perpendicular/  1 to 1.3 cun |
| Guanyuan (CV04) | 3 cun below the umbilicus. | Perpendicular/  1 to 1.3 cun |
| Shangjuxu (ST37) | 6 cun below Dubi (ST35), one finger-breadth lateral to the anterior border of the tibia. | Perpendicular/  1 to 1.3 cun |

1 cun (≈25 mm) is defined as the width of the interphalangeal joint of the patient's thumb

4.3 Control group (Sham acupuncture group)

4.3.1 Acupuncture preparation

(1) Sterile flat-head acupuncture needles (0.30 mm×10 mm, Guangzhou Suixin Medical Supplies Co., Ltd., Guangzhou, China); (2) Sham acupuncture devices without an aperture (Guangzhou Suixin Medical Equipment Co., Ltd., Guangzhou, China).

4.3.2 Acupoints selection

The acupoints will be the same as in the MA group.

4.3.3 Operation method

The patients will be placed in a supine position and wear an eye patch. The physicians will precisely locate the acupoints according to the standard, routinely disinfect the skin of the patients, paste the sham acupuncture device, adjust the cannula according to the application site, and use the flat-head acupuncture needles without piercing the skin. All acupoints will be left in place for 30 minutes before being removed, and the needle holes will be pressed for a few moments with a sterilized, dry cotton swab when the needles are out. All patients will receive 12 sessions of treatment, three times a week (Tuesday, Thursday, and Saturday), for a period of four weeks.

4.4 Emergency treatment

When patients don`t have bowel movements for more than three consecutive days or experience intolerable symptoms such as bloating due to constipation, they are allowed to use emergency medications under medical supervision with detailed documentation. The emergency treatment consisted of lactulose and glycerin enemas. Lactulose will be used for patients who do not have bowel movements, and glycerin enemas will be used for patients who strain during defecation. The specifications of the emergency medications are as follows: Lactulose Oral Liquid (Hunan Keren Pharmaceutical Company, Approval No.: State Pharmaceutical Code H20093523), 15 ml per dose at 12-hour intervals, 2 doses in total; Glycerin Enemas (Guangdong Hengjian Pharmaceutical Company, Approval No.: H44023815), 1 stick each time in total.

5. Statistical analysis and processing

SPSS 26.0 will be used as the statistical analysis software for this study. We used the intention-to-treat (ITT) principle, regardless of whether the patients completed all assigned intervention sessions. Continuous variables were expressed as mean (SD) or median (IQR); independent t-tests were used for normally distributed values, and Mann-Whitney U tests were used for skewed data. Categorical variables were expressed as frequencies and percentages and analyzed using the χ2 or Fisher exact test, as appropriate. A repeated measures analysis of variance (ANOVA) was used to detect trends in score changes and treatment effects at different time points within groups. Multiple interpolation (MI) methods were used to estimate missing data for the primary and secondary outcomes. To assess the robustness of MI methods under the assumption of randomization, sensitivity analyses of the primary outcome were performed using per-protocol (PP) sets. Differences between groups were reported using 95% confidence intervals and bilateral P values, with values less than 0.05 considered significant.

Safety analysis: Adverse events are analyzed in all participants who received treatment, including the incidence of adverse events and the specific description of adverse events. The data are summarized as n (%). χ2 test or Fisher’s exact test is used for the comparison between treatment groups.

Blinding analysis: The blinding analysis will be performed on all included study cases. The data are summarized as n (%). For categorical variables, χ2 test or Fisher’s exact test is used for the comparison between treatment groups.

Analysis of dropouts: description of dropout cases, including their causes, calculation of the proportion of dropout cases to the total number, and the incidence of dropouts. The data are summarized as n (%). χ2 test or Fisher’s exact test is used for the comparison between treatment groups.

6. Quality Control

Before the study, each patient will be instructed to keep a bowel symptom diary. To ensure accurate and complete data recording, they will be reminded and supervised throughout the study. All patients will be assessed 4 hours after taking their anti-Parkinson drugs. Primary outcomes will be collected electronically, and secondary outcomes will be assessed on paper and organized in Excel.

7. Ethical Approval

This study protocol has been approved by the Ethics Committee of The First Affiliated Hospital of Guangzhou University of Chinese Medicine.

8. Informed Consent

Project name: Observation of clinical efficacy of acupuncture for the treatment of Parkinson's disease-related constipation (PDC)

Clinical trial institution: The First Affiliated Hospital of Guangzhou University of Chinese Medicine

**Personal reading materials**

Dear patients, please read this article carefully, and you are welcome to ask questions and discuss it with us or your family, relatives, and friends.

You are invited to participate in this clinical study. The purpose of the study is to prove the clinical efficacy of acupuncture for the treatment of Parkinson's disease-related constipation by comparing acupuncture with sham acupuncture and to provide technical and evidence-based medicine for the treatment of Parkinson's disease-related constipation.

Whether to participate in this study depends entirely on your wishes; please read this material in detail before you decide. It assists you in developing a thorough understanding of the study's purpose, methods, research process, potential benefits, and drawbacks. The information provided to you by this informed consent form can help you decide whether to participate in this clinical trial. Please ask any questions from the investigator responsible for the project trial or discuss it with your family, relatives, and friends to ensure that you fully understand the content. Whether you participate in this trial is voluntary, and if you agree to participate in this clinical trial, please sign the statement in the informed consent form.

**1、What kind of study is this? [Research Background and Purpose]**

This study is entitled "Clinical Efficacy of Acupuncture for the Treatment of Parkinson's Disease-Related Constipation." The study compares the clinical efficacy of the two therapies for Parkinson's disease-related constipation, manual acupuncture combined with anti-Parkinson medications and sham acupuncture combined with anti-Parkinson medications, and provides a technical and evidence-based medicine basis for the treatment of Parkinson's disease-related constipation.

In the previous clinical study, the effect of acupuncture on the improvement of constipation in Parkinson's disease (PD) patients was preliminary found by the increase of weekly complete spontaneous bowel movements (CSBMs). The effect of acupuncture on the improvement of motor symptoms in PD patients was preliminary found by the UPDRS.

In this study, the cases in the outpatient department of Parkinson's disease at the First Affiliated Hospital of Guangzhou University of Chinese Medicine are collected from May 2022 to November 2022 and in strict accordance with the ethical review. The total number of cases are 78, with 39 cases in each group. The patients' original anti-Parkinson medication dose is maintained from before the start of treatment until the end of treatment. The efficacy of the treatment is evaluated using the scores of weekly complete spontaneous bowel movements (CSBMs), the constipation symptom and efficacy assessment scale (CSEAS), the constipation patient quality of life scale (PAC-QOL), and the UPDRS scale before and at the end of treatment, respectively. The clinical advantages of acupuncture in the treatment of Parkinson's disease-related constipation are investigated from different perspectives.

**2、Is participation in this study voluntary?**

Participation in this study is voluntary. You have the right to decide whether to participate in this study without giving any reason. Failure to participate in this study will not subject you to any discrimination or retaliation; it will not affect your relationship with your doctor or your medical interests, and you will continue to receive the treatment given by your doctor.

**3、Who should not participate in this project research?**

You will not be eligible for this study if you have any of the following conditions:

(1) Noncompliance with inclusion criteria.

(2) Having organic lesions of the digestive system (such as intestinal adhesions, obstruction, tumors, or malformations in the gastrointestinal tract, etc.), or a history of abdominal or anorectal surgery that may affect intestinal transit, or systemic diseases that affect the dynamics of the digestive tract (such as diabetes mellitus, hyperthyroidism, etc.).

(3) Serious life-threatening diseases such as severe cardiovascular and malignant tumors, etc.

(4) Those who have skin lesions that are not inappropriate for needling, or those who have viscose allergy that prevent acupuncture device attachment.

(5) Pregnant or lactating women.

**4、What will you need to do if you participate in the study?**

If you participate in this study, you will enter the screening period after signing the informed consent form. If your doctor thinks that you meet the inclusion criteria and are suitable to participate in this study, you will receive treated based on the random number generated by the computer. You will have 50% chance to enter the manual acupuncture group and 50% chance to enter the sham acupuncture group.

**5、Possible benefits of participating in the study?**

You and the community may or may not benefit directly from this study. Such benefits include the potential for improvement in your condition and this study may help further clarify the value of acupuncture in the treatment of Parkinson's disease-related constipation for the benefit of patients with conditions like yours.

**6、Possible adverse reactions, risks, discomfort, and inconvenience of participating in the study**

The following are some of the potential side effects of participating in the study: halo needles, blocked needles, bent needles, broken needles, bleeding and subcutaneous hematomas, peripheral nerve injuries, and so on. In the event of an adverse reaction, the researcher will discontinue the current treatment and take the necessary treatment measures. Meanwhile, the medical expenses caused by the adverse reactions will be borne by the research group.

**7、Related fees**

The research team will pay your costs related to the study, including: acupuncture treatment costs, treatment equipment (needles, medical cotton swabs) and traffic fees.

**8、How do you protect your privacy rights?**

Your medical records (including research medical records, etc.) will be kept in the hospital as required. Your participation in the study and your personal information in the study are confidential, and the results of the study will not reveal your personal identity. Health, drug, and research administration, hospital ethics committee, and investigators will be allowed to access your medical records to verify procedures and data for the clinical study. We will strictly protect the privacy of your personal medical information within the existing laws.

**9、Important tips**

In order to ensure the reliability of clinical research, I hope you can do the following throughout the clinical research: ① Do not receive other similar treatment; ② Do not self-administer gastrointestinal motility medications.③ For any discomfort during research, please report it to your doctor in charge.

The treatment plan for this study is not the only treatment option for your current disease, so you can consult with your doctor and then decide whether to participate in this study.

**10、Can you quit after attending the study?**

It is entirely up to you whether or not you attend. You may refuse to participate in this study or withdraw at any time during the course of the study, neither affecting your relationship with your doctor nor resulting in the loss of your medical or other interests, and you are free from any discrimination or retaliation. Your doctor may suspend your participation in this study at any time in your best interests. If you withdraw from the study for any reason, in your best interest, you may be asked about your medication use. If you choose to participate in this study after full consideration, we want you to complete the whole study process.

**11、More information acquisition**

You can ask any questions about this study at any time, and your doctor will leave you with contact information to allow you to answer your questions.

Your doctor will inform you promptly if there is any important new information during the study that may affect your willingness to continue attending the study.

Finally, thank you for reading the above materials. If you decide to participate in this study, please tell your doctor that they will arrange for you to participate in it. Please keep this information on file. If you have any questions about your rights in this study, please contact your doctor.

Statement of Subject

I have carefully read this informed consent form, and I have had the opportunity to ask questions, and all questions have been answered. I understand that participation in this study is voluntary, and I can choose not to participate in it or withdraw at any time after notifying the researcher without being discriminated against or retaliated against, and my medical treatment and rights will not be affected.

If I require additional diagnosis or treatment, if I do not follow the trial schedule, or if there are other reasonable reasons, the investigator may terminate my participation in the clinical study.

Subject's Signature: Date: Year/month/day

Contact Number:

Signature of Legal Agent [if applicable] :

Relationship with subject: Date: Year/month/day

Statement of the Researcher

I have accurately informed the subject of the informed consent and answered their questions. The subject is willing to participate in this clinical study.

Investigator's Signature: Date: Year, month, day

Contact Number:

9. References

Barboza, J.L., Okun, M.S., and Moshiree, B. (2015). The treatment of gastroparesis, constipation and small intestinal bacterial overgrowth syndrome in patients with Parkinson's disease. *Expert Opin Pharmacother* 16(16)**,** 2449-2464. doi: 10.1517/14656566.2015.1086747.

Bloem, B.R., Okun, M.S., and Klein, C. (2021). Parkinson's disease. *Lancet* 397(10291)**,** 2284-2303. doi: 10.1016/s0140-6736(21)00218-x.

Chou, K.L., Stacy, M., Simuni, T., Miyasaki, J., Oertel, W.H., Sethi, K., et al. (2018). The spectrum of "off" in Parkinson's disease: What have we learned over 40 years? *Parkinsonism Relat Disord* 51**,** 9-16. doi: 10.1016/j.parkreldis.2018.02.001.

Gan, J., Wan, Y., Shi, J., Zhou, M., Lou, Z., and Liu, Z. (2018). A survey of subjective constipation in Parkinson's disease patients in shanghai and literature review. *BMC Neurol* 18(1)**,** 29. doi: 10.1186/s12883-018-1034-3.

Lesser, G.T. (2002). Frequency of bowel movements and future risk of Parkinson's disease. *Neurology* 58(5)**,** 838; author reply 838-839. doi: 10.1212/wnl.58.5.838-a.

Li, K., Wang, Z., Chen, Y., Shen, L., Li, Z., Wu, Y., et al. (2019). Efficacy of electroacupuncture for the treatment of constipation in Parkinson's disease: study protocol for a multicentre randomised controlled trial. *BMJ Open* 9(11)**,** e029841. doi: 10.1136/bmjopen-2019-029841.

Li, Q., Wu, C., Wang, X., Li, Z., Hao, X., Zhao, L., et al. (2022). Effect of acupuncture for non-motor symptoms in patients with Parkinson's disease: A systematic review and meta-analysis. *Front Aging Neurosci* 14**,** 995850. doi: 10.3389/fnagi.2022.995850.

Mozaffari, S., Nikfar, S., Daniali, M., and Abdollahi, M. (2020). The pharmacological management of constipation in patients with Parkinson's disease: a much-needed relief. *Expert Opin Pharmacother* 21(6)**,** 701-707. doi: 10.1080/14656566.2020.1726319.

Nazarova, L., Liu, H., Xie, H., Wang, L., Ding, H., An, H., et al. (2022). Targeting gut-brain axis through scalp-abdominal electroacupuncture in Parkinson's disease. *Brain Res* 1790**,** 147956. doi: 10.1016/j.brainres.2022.147956.

Palsson, O.S., Whitehead, W.E., van Tilburg, M.A., Chang, L., Chey, W., Crowell, M.D., et al. (2016). Rome IV Diagnostic Questionnaires and Tables for Investigators and Clinicians. *Gastroenterology*. doi: 10.1053/j.gastro.2016.02.014.

Postuma, R.B., Berg, D., Stern, M., Poewe, W., Olanow, C.W., Oertel, W., et al. (2015). MDS clinical diagnostic criteria for Parkinson's disease. *Mov Disord* 30(12)**,** 1591-1601. doi: 10.1002/mds.26424.

Schapira, A.H.V., Chaudhuri, K.R., and Jenner, P. (2017). Non-motor features of Parkinson disease. *Nat Rev Neurosci* 18(7)**,** 435-450. doi: 10.1038/nrn.2017.62.

Wang, Y.T., Liu, X., Xu, Z.Q., and Zhuang, L.X. (2022). [An auxiliary device for double-blind placebo acupuncture research]. *Zhongguo Zhen Jiu* 42(3)**,** 351-354. doi: 10.13703/j.0255-2930.20210227-k0001.
