# Supplementary material for: Efficacy of acupuncture for the treatment of Parkinson’s disease-related constipation (PDC): A randomized controlled trial
Source: Front Neurosci. 2023 Feb 13;17:1126080. doi: 10.3389/fnins.2023.1126080 (PMC9972583; doi:10.3389/fnins.2023.1126080)
Supplement: Supplementary file 2 [file Data_Sheet_2.docx]

Supplementary 2

Table S1 Primary outcome measurements of the per-protocol (PP) population

|  | MA group (n=36) | SA group (n=35) | Difference (95% CI) | *P* |
| --- | --- | --- | --- | --- |
| Weekly CSBMs, mean (SD) | | | | |
| Posttreatment (week 4) | 4.67(1.90) | 3.09(1.25) | 1.58(0.82 to 2.34) | ＜.001 |
| Follow-up (week 8) | 4.28(1.41) | 3.06(1.28) | 1.22(0.58 to 1.86) | ＜.001 |

|  | Mean change from baseline (95% CI) |  | Mean change from baseline (95% CI) |  |
| --- | --- | --- | --- | --- |
| Variable | MA group (n=36) | *P* | SA group (n=35) | *P* |
| Weekly CSBMs | | | | |
| Posttreatment (week 4) | 1.42(0.92 to 1.92) | ＜.001 | -0.03(-0.33 to 0.28) | 0.851 |
| Follow-up (week 8) | 1.03(0.66 to 1.39) | ＜.001 | -0.06(-0.33 to 0.22) | 0.676 |

Table S2 Details of adverse events

|  | MA group (n=39) | SA group (n=39) |
| --- | --- | --- |
| Adverse events | 6(15.4) | 0(0.0) |
| Bleeding, No. (%) | 3(7.7) | 0(0.0) |
| Subcutaneous hematoma, No. (%) | 2(5.1) | 0(0.0) |
| Sharp pain, No. (%) | 1(2.6) | 0(0.0) |

Table S3 Assessment of Blinding

|  | MA group (n=39) | SA group (n=39) | *P* |
| --- | --- | --- | --- |
| Blinding assessments |  |  | 0.276 |
| Pierced acupuncture, No. (%) | 28(71.8) | 26(66.7) |  |
| Non-pierced acupuncture, No. (%) | 3(7.7) | 4(10.3) |  |
| Don’t know, No. (%) | 8(20.5) | 9(23.1) |  |
