# Supplementary material for: Efficacy of acupuncture for the treatment of Parkinson’s disease-related constipation (PDC): A randomized controlled trial
Source: Front Neurosci. 2023 Feb 13;17:1126080. doi: 10.3389/fnins.2023.1126080 (PMC9972583; doi:10.3389/fnins.2023.1126080)
Supplement: Supplementary file 3 [file Data_Sheet_3.docx]

Statement

Before the start of the clinical study, we discovered the clinical protocol registered in the Chinese Clinical Trial Registry was imperfect; hence, we modified it to best reflect the efficacy of acupuncture in treating Parkinson's disease-related constipation. The revisions are as follows:

| Revision | Before | After | Reason |
| --- | --- | --- | --- |
| Age of inclusion criteria | Age between 35 and 75 years | Age between 35 and 80 years | We discovered that many Parkinson's disease-related constipation patients in clinical were between the ages of 70 and 80. We raised the upper age restriction in the inclusion criteria to 80 years in order to avoid bias caused by enrolling patients who are too young in the experiment. |
| Blinding | Blinding of outcome assessors and statisticians only. | Patients, outcome assessors, and statisticians were all blinded. | Credible blinding was an essential part of high-quality research. A well-designed sham acupuncture device was designed by our team members, which ensured physiologically inert blinding. |
| Grouping | There were four groups in total, including the basic treatment group, and three acupuncture groups with different combinations of acupuncture points. | There were two groups, including the manual acupuncture group, and the sham acupuncture group. | The first protocol grouping was flawed since patients were not blinded to rule out the acupuncture placebo effect and comparisons between groups did not evaluate the clinical effect of acupuncture for constipation in Parkinson's disease. To implement a placebo control, we reset the groupings and refined the blinding method. |
| Secondary outcomes | Bristol Stool Form Scale and the Patient Assessment of Constipation-Symptoms (PAC-SYM) | The Constipation Symptom and Efficacy Assessment Scale (CSEAS) | CSEAS comprises six questions covering six constipation-related symptoms. It included the Bristol Stool Form Scale. And its succinct question reflects the efficiency of the treatment in a comprehensive and targeted way, which was more suitable for Parkinson's disease-related constipation patients. |
