# Supplementary figures and images for: Efficacy of acupuncture for the treatment of Parkinson’s disease-related constipation (PDC): A randomized controlled trial
Source: Front Neurosci. 2023 Feb 13;17:1126080. doi: 10.3389/fnins.2023.1126080 (PMC9972583; doi:10.3389/fnins.2023.1126080)

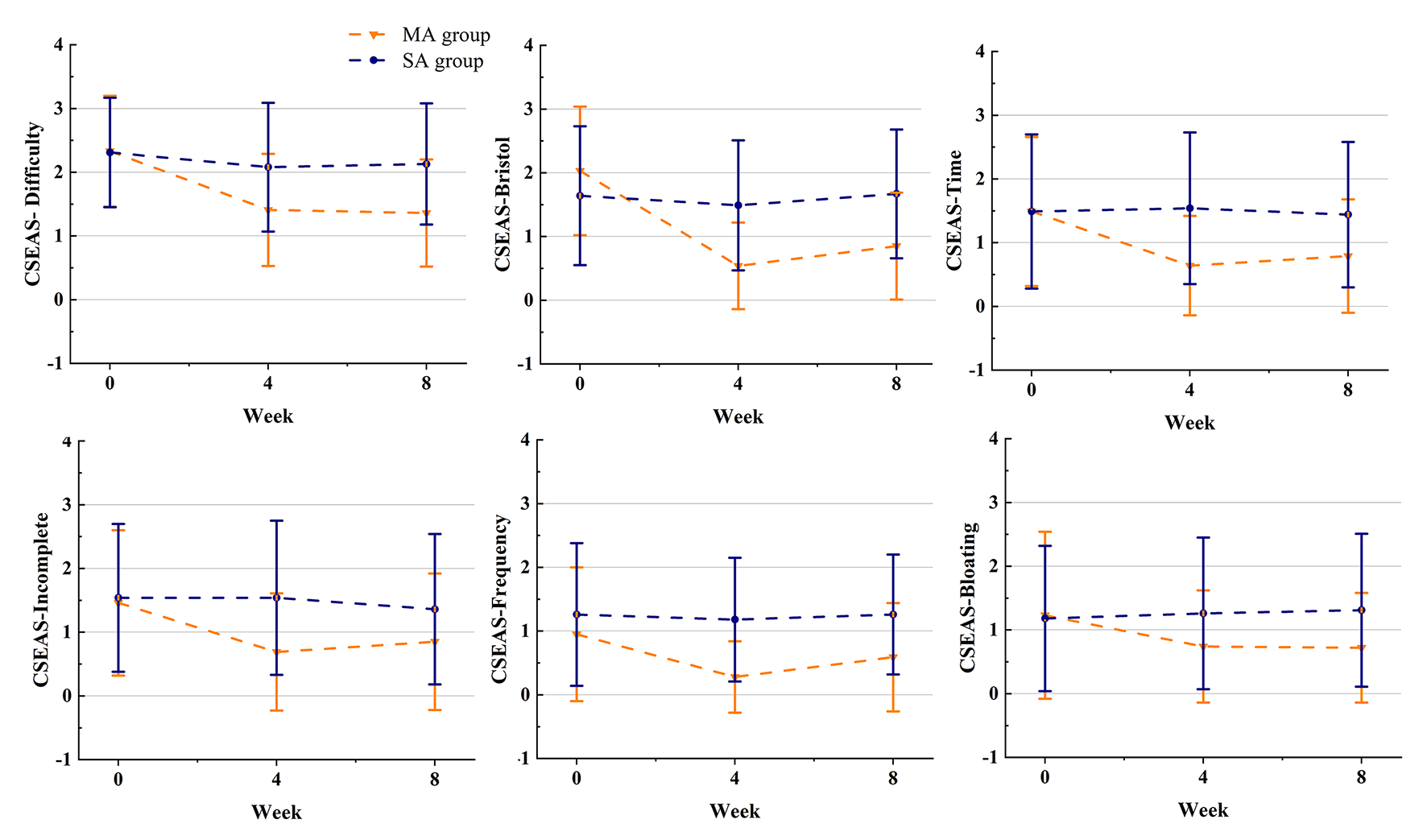

Supplement: Supplementary file 4 [file Image_1.TIF]
